# Comparative Genomic Analysis of Two *Phyllanthus emblica* Genomes with Endemic and Widespread Cultivar Backgrounds

**DOI:** 10.3390/life16071138

**Published:** 2026-07-09

**Authors:** Yongqin Zheng, Qinghan Wu, Yuzhong Zheng, Jianjian Huang, Fengnian Wu

**Affiliations:** 1 School of Life Sciences and Food Engineering, Hanshan Normal University, Chaozhou 521041, China; zhengyongqin332@gmail.com (Y.Z.); 2078@hstc.edu.cn (Q.W.); zhengyuzhon@hstc.edu.cn (Y.Z.); 2 Guangdong Key Laboratory of Functional Substances and Health Products from Medicinal Edible Resources, Hanshan Normal University, Chaozhou 521041, China

**Keywords:** *Phyllanthus emblica*, whole-genome sequencing, molecular markers

## Abstract

*Phyllanthus emblica* is valued for its nutritional and medicinal properties, yet the genomic divergence between localized and widespread cultivars remains poorly understood. We investigated the genomes of two individuals from the endemic cultivar ‘Hongguang’ (HG), propagated via regional grafting, and the commercially widespread ‘Dongkeng’ (DK), known for its superior protein content. Using whole-genome sequencing, we reconstructed phylogenies from two nuclear markers, profiled genome-wide variations, assembled chloroplast genomes, and verified relative plastid copy numbers via real-time quantitative PCRs (qPCRs). Nuclear internal transcribed spacer (ITS) and phytochrome C (*PHYC*) phylogenies confirmed both samples belonged to the *P. emblica* lineage, while revealing a distinct genetic identity for the HG individual. Genome-wide variant profiling of the two individuals identified KEGG enrichment in plant hormone signaling pathways; DK variants mapped to the canonical auxin axis, while HG variants were annotated to reversible protein phosphorylation. Comparative chloroplast genomics demonstrated shared maternal inheritance and shared mutations in key photosynthetic genes (*psaB*, *petA*, and the *ndh* cluster) between the two genomes, though qPCR validation revealed a higher relative chloroplast DNA copy number in the DK sample. Despite the two-individual limitation, these findings revealed preliminary genomic variations, offering candidate molecular markers for future population studies and marker-assisted breeding.

## 1. Introduction

Amla (*Phyllanthus emblica* L., synonym *Emblica officinalis*), a deciduous tree belonging to the family Phyllanthaceae, is native to Southeast Asia and is currently widely distributed throughout tropical and subtropical regions, including China, Indonesia, and Malaysia [1]. Highly valued for both its nutritional and medicinal properties, the fruit of *P. emblica* serves as a rich source of vitamin C and polyphenols, making it an important resource in both the food and pharmaceutical industries [2]. Moreover, due to its diverse antioxidant, anti-inflammatory, and immunomodulatory bioactivities, *P. emblica* is documented in traditional medicine for the clinical management of chronic disorders such as diabetes, dyslipidemia, and liver diseases [3]. Recently, biological research on *P. emblica* has shifted from agronomic trait evaluations to molecular assessments, expanding our understanding of its genetic structure through sequencing data and the establishment of expressed sequence tag–simple sequence repeat (EST-SSR) markers linked to major metabolic pathways for future breeding programs [4]. However, the complex ploidy of *P. emblica*, including diploid, polyploid, or mixed status, often restricts the efficacy of conventional SSR markers, while classic morphological traits remain confounded by environmental and edaphic variations, hindering precise lineage evaluation [5]. Therefore, systematically establishing fine-scale molecular profiling across diverse germplasms is crucial for genetic resource conservation and standardized cultivation.

With the advancement of high-throughput sequencing technologies, organelle and nuclear genomics have emerged as powerful tools for resolving phylogenetic relationships and identifying functional variations in *Phyllanthus* species, marked by the recent chromosome-level genome assembly of *P. emblica* that uncovered specific metabolic pathways responsible for its super antioxidant properties and flavonoid accumulation [6]. Subsequently, the chromosome-scale genome assembly of the *P. emblica* wild-type ‘Yingyu’ decoded the complex octoploid genomic landscape and highlighted key candidate genes regulating ascorbate biosynthesis, providing an indispensable genomic anchor for deep comparative analyses and elite cultivar selection [7]. Complementing nuclear genomics, de novo chloroplast genome assemblies within *Phyllanthus* revealed specific genomic characteristics, such as *rps16* pseudogenization and highly polymorphic functional genes, expanding the toolkit for high-resolution systematic inference and maternal lineage tracking [8]. Despite these advancements, the genomic stratification and micro-evolutionary divergence driving different commercial and endemic cultivars remain poorly understood. For instance, while the widely cultivated cultivar ‘Dongkeng’ (DK) is favored in agriculture for its exceptional protein content [9], the localized cultivar ‘Hongguang’ (HG) represents a unique, endemic germplasm propagated through regional grafting [10]. To date, a comparative framework evaluating the dual nuclear and chloroplast mutational landscapes of these distinct lineages has been lacking. To address this gap, we performed whole-genome sequencing and *de novo* chloroplast assembly on two specific individuals with DK and HG cultivar backgrounds. By authenticating taxonomic lineages, predicting variant-enriched pathways through KEGG functional annotation, and identifying chloroplastic variations, we explored the genomic and evolutionary differences between the two samples. While limited to a two-sample comparison, these findings provided candidate genomic resources for future marker-assisted breeding and germplasm conservation.

## 2. Materials and Methods

### 2.1. Plant Materials and DNA Sequencing

Fresh leaves of the endemic *Phyllanthus emblica* cv. ‘Hongguang’ (designated as HG), morphologically characterized by its larger fruit weight and diameter, were collected from Hongguang Village, Jieyang City, Guangdong Province, China (23.3862 °N, 115.9982 °E). This cultivar originated from a wild individual identified in 2007 and has since been propagated via local grafting [10]. For comparison, leaves of the widely cultivated *P. emblica* cv. ‘Dongkeng’ (designated as DK) were obtained from the Shantou Forestry Research Institute, Guangdong, China. This cultivar features a long cultivation history with an unknown discovery date, and it is highly valued in the food industry for its superior protein content despite its relatively smaller fruit size [9]. Photographs of the DK and HG samples are available at Figshare (DOI: 10.6084/m9.figshare.32625522). Prior to DNA extraction, healthy leaves were surface-sterilized with absolute ethanol. For each sample, 0.1 g of fresh tissue was ground in liquid nitrogen, and total genomic DNA was extracted using the standard CTAB method. DNA concentration was quantified using the Qubit 3.0 fluorometer (Invitrogen, Carlsbad, CA, USA). For high-throughput sequencing, 1 μg of genomic DNA per sample was utilized to construct a 350 bp short-insert Ultra DNA library (NEB, Ipswich, MA, USA), and 150 bp sequencing was performed on the Illumina HiSeq 2000 platform (Illumina, San Diego, CA, USA). After quality control using fastp v.1.0.1 [11], the clean reads were deposited (NGDC BioSample accessions: SAMC6851733 (DK) and SAMC6851734 (HG)).

### 2.2. Nuclear Phylogenetic Analysis

The phylogenetic reconstruction and variant calling were performed using CLC Genomics Workbench v.20.0.4. To verify the taxonomic identities of samples DK and HG, the nuclear internal transcribed spacer (ITS) region and phytochrome C (*PHYC*) gene were employed as molecular markers, and sequences from *P. emblica* and five closely related *Phyllanthus* species were retrieved as references, including *P. annamensis*, *P. geoffrayi*, *P. oxyphyllus*, *P. phuquocensis*, and *P. racemosa* (Tables S1 and S2) [12]. The ITS- and *PHYC*-related reads from DK and HG were extracted via reference-based mapping (Length fraction = 0.8, Similarity fraction = 0.8), followed by de novo assembly (Minimum contig length = 200 bp) to generate target contigs (NGDC GenBase accessions: C_AA158584 (DK_ITS), C_AA158585 (HG_ITS), C_AA158586 (DK_PHYC), C_AA158587 (HG_PHYC)). A total of 22 (ITS) and 11 (*PHYC*) sequences were aligned respectively, and maximum likelihood (ML) phylogenetic trees were reconstructed (Model = Hasegawa-Kishino-Yano, Bootstrap = 1000).

### 2.3. Variant Calling and Functional Annotation

For variant calling, the genome of the *P. emblica* wild-type ‘Yingyu’ served as the reference [7]. Single-nucleotide polymorphisms (SNPs) and small insertions/deletions (InDels) were identified via reference-based mapping (Length fraction = 0.9, Similarity fraction = 0.9, Minimum frequency: 35%, Minimum coverage: 10) (NGDC genome variation map accession: GVM001319). After filtering out shared variants between the two samples, the remaining sample-specific variants were categorized into non-coding regions or coding sequences (CDS) based on the *P. emblica* reference genome annotation (Table S3). The variant-related genes were functionally annotated using eggNOG-mapper v.2.1.13 [13] and categorized into KEGG pathways using the clusterProfiler v.4.18.4 package in R v.4.2.0 [14]. After excluding prokaryotic and animal-related pathways, the top 10 enriched metabolic pathways were selected to identify sample-specific variant genes (Table S4).

### 2.4. Chloroplast Genome Assembly and Comparison

The chloroplast genome assembly was performed via NOVOPlasty v.4.3.5 (K-mer = 39) using clean reads [15], and the *trnK*-*matK* gene (2604 bp) from the *P. emblica* chloroplast genome (GenBank accession: MN122078) was employed as the seed sequence. Subsequently, reference-based mapping was conducted in CLC Genomics Workbench v.20.0.4 (Length fraction = 1.0, Similarity fraction = 1.0) to confirm the absence of mismatches. To confirm the circularity of the genome, reads were mapped across the 2000 bp junction of the joined ends, finalizing the complete circular sequence. The chloroplast genome annotation was performed using the GeSeq server (NGDC GenBase accessions: C_AA159610 (DK_chl), C_AA159611 (HG_chl)) [16]. A genome-wide ML phylogenetic tree (Model = General reversible model (GTR), Bootstrap = 1000) was constructed using CLC Genomics Workbench v.20.0.4 for *P. emblica* and seven related species, including *P. acidus*, *P. amarus*, *P. franchetianus*, *P. niruri*, *P. pulcher*, *P. reticulatus*, *P. urinaria* (Table S5) [17]. A Bayesian divergence time tree was reconstructed using MCMCTree v.4.9 with an independent rates model [18], employing the divergence time of 13.5–35.4 million years ago (Ma) between *P. acidus* and *P. amarus* as the calibration node [19]. Comparative genomic analysis between the DK and HG chloroplasts was performed using snippy v.4.6.0 (https://github.com/tseemann/snippy, accessed on 7 July 2026) referenced against the *P. emblica* sequence (GenBank accession: NC_047477), and vcf-annotator v.0.7 was employed to identify variants located in CDS (https://github.com/rpetit3/vcf-annotator, accessed on 7 July 2026) (Table S6).

### 2.5. Real-Time Quantitative PCR (qPCR)

To identify chloroplast-specific sequences, the unmapped reads filtered from the chromosome-based mapping were re-mapped to the *P. emblica* chloroplast genome, and the resulting covered regions (>10×) were identified as chloroplast-specific fragments. To determine the relative chloroplast copy number, qPCR was conducted for each cultivar in three biological replicates using the chromosome-specific primers (PHYC_F: 5′-TGTGCTATGTGACATGCTCC-3′, PHYC_R: 5′-GTGCAGCTCCATCACACTTA-3′) and chloroplast-specific primers (rps16_F: 5′-AGTCAAGAGCACCTCCACTC-3′, rps16_R: 5′-AGGACATGATCGGTTGTGGA-3′). Primers were designed via Primer3web v.4.1 (https://primer3.ut.ee/) (Product size range = 90–100 bp). qPCR was conducted on the CFX connect real-time PCR detection system (Bio-Rad, Hercules, CA, USA) in a total volume of 20 μL, containing 10 μL of Bestar Sybr green qPCR master mix (DBI Bioscience, Newark, DE, USA), 0.5 μL of each primer, 1 μL of DNA template (~50 ng), and 8 μL of ddH_2_O. The amplification program was performed with an initial denaturation at 90 °C for 5 min, followed by 40 cycles of 90 °C for 5 s and 65 °C for 30 s, with fluorescence signals recorded at the end of each cycle to determine the threshold cycle (Ct) values based on automated baseline and threshold settings. The relative chloroplast copy number was calculated using the 2^−ΔCt^ method (ΔCt = Ct (rps16) − Ct (PHYC)) [20].

## 3. Results and Discussion

### 3.1. Nuclear Phylogeny Revealed the Genetic Distinctiveness of Two Phyllanthus emblica Genomes

**Figure 1:**
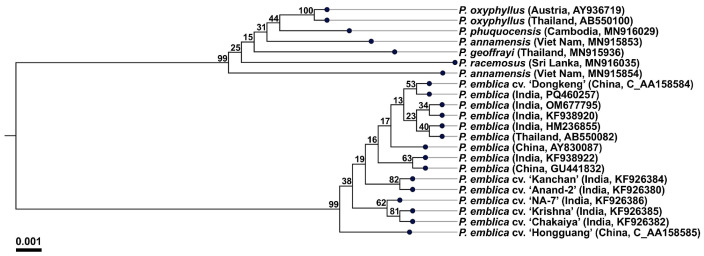
The maximum likelihood (ML) phylogenetic tree of *Phyllanthus emblica* cv. ‘Dongkeng’ (DK) and ‘Hongguang’ (HG) and five closely related *Phyllanthus* species based on the nuclear internal transcribed spacer (ITS) region.

**Figure 2:**
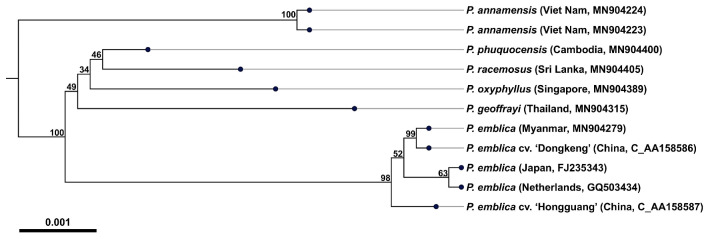
The maximum likelihood (ML) phylogenetic tree of *Phyllanthus emblica* cv. ‘Dongkeng’ (DK) and ‘Hongguang’ (HG) and five closely related *Phyllanthus* species based on the phytochrome C (*PHYC*) gene.

After quality control, a total of 279,872,079 and 284,078,331 clean reads were obtained for the sampled individuals of *P. emblica* cv. ‘Dongkeng’ (DK) and cv. ‘Hongguang’ (HG), respectively. To authenticate the taxonomic identities, sequences of the nuclear internal transcribed spacer (ITS, 426–427 bp) and the phytochrome C (*PHYC*, 567 bp) gene were extracted for phylogenetic reconstruction (Tables S1 and S2). Compared with the high coverage of the multicopy ITS region (8397.00–10,460.00×), the *PHYC* gene exhibited a much lower coverage (49.85–79.69×) that closely matched the average genome-wide sequencing depth (83.52–84.67×) (Tables S1 and S2), suggesting that *PHYC* remained a single-copy gene in *P. emblica*, as documented in other angiosperms [21]. The maximum likelihood (ML) analysis confirmed that both the DK and HG samples clustered within the *P. emblica* lineage (Figure 1 and Figure 2). The ITS-based phylogeny showed that all *P. emblica* accessions were phylogenetically distant from other examined *Phyllanthus* species (Figure 1). Consistently, the *PHYC*-based tree revealed that *P. emblica* lineage was most closely related to *P. geoffrayi* and most divergent from *P. annamensis*, a finding that aligned with previous systematic frameworks (Figure 2) [12]. This topological discrepancy suggested that the *PHYC* gene provided superior resolution for resolving phylogenetic relationships among subgeneric lineages within *Phyllanthus*. Within the *P. emblica* cluster, both markers indicated that sample HG was relatively independent from other accessions, including DK (Figure 1 and Figure 2), highlighting the unique genetic identity of the sequenced ‘Hongguang’ individual. This distinctiveness was congruent with recent findings demonstrating significant genomic stratification within *P. emblica* populations, which were structured into discrete clusters that potentially reflect the evolutionary divergence between wild and commercial lineages [22]. Importantly, these results represented preliminary genetic profiles rather than population-wide cultivar characteristics, and broader sampling will be necessary to confirm lineage distinctiveness.

### 3.2. Variant Profiling and Functional Dissection of Signaling Pathways in Two Phyllanthus emblica Genomes

A variant calling analysis, using the *P. emblica* ‘Yingyu’ genome as a reference, revealed substantial genomic divergence between the two sequenced individuals. A total of 3,458,532 and 3,434,313 variants were identified in the DK and HG samples, respectively (Table S3). The vast majority of these variants were located in non-coding regions (96.99% in DK, 97.00% in HG), while a smaller but substantial proportion of coding variants was identified (3.01% in DK, 3.00% in HG) (Table S3), demonstrating a consistent variant distribution profile between the two genomes. Although limited to two specific samples, the identified extensive genomic polymorphisms provided candidate markers for reliable cultivar differentiation, overcoming the environmental and edaphic confounding typical of traditional morphological traits [23].

**Figure 3:**
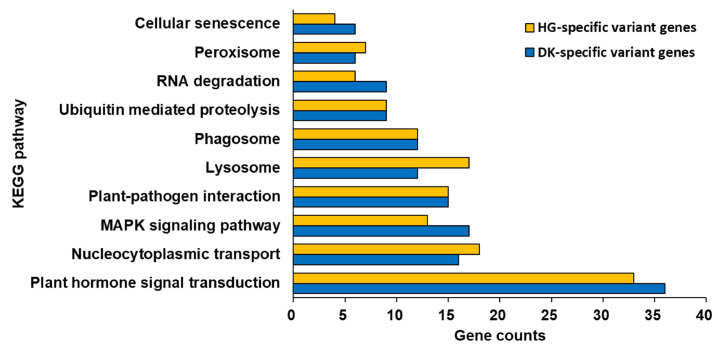
The top 10 KEGG functional pathways enriched with variant-related genes. The bar chart illustrated the number of genes containing sample-specific variants in *Phyllanthus emblica* cv. ‘Dongkeng’ (DK) and ‘Hongguang’ (HG) for each metabolic category.

Functional annotation and KEGG enrichment analysis of genes harboring sample-specific variants revealed that the top 10 biological pathways could be categorized into three functional modules: signal transduction (“Plant hormone signal transduction”, “MAPK signaling pathway”, “Plant-pathogen interaction”), intracellular homeostasis (“Nucleocytoplasmic transport”, “Ubiquitin mediated proteolysis”, “RNA degradation”), and cellular fate (“Lysosome”, “Phagosome”, “Peroxisome”, “Cellular senescence”) (Table S4, Figure 3). Notably, previous research in *P. amarus* demonstrated that endogenous hormone levels and their dynamic ratios, particularly between auxins (AUXs) and cytokinins (CKs), which may alter the sensitivity or efficiency of phytohormone signaling, play a pivotal role in organ development [24]. Furthermore, prior research highlighted that the mitogen-activated protein kinase (MAPK) served as a central signaling hub that activated downstream transcription factors (such as WRKYs) and stress-responsive genes during environmental adaptation [25]. Correspondingly, the variants identified within the “Plant hormone signal transduction” and “MAPK signaling pathway” in the two genomes could modulate their signaling efficiency and downstream cellular responses.

**Figure 4:**
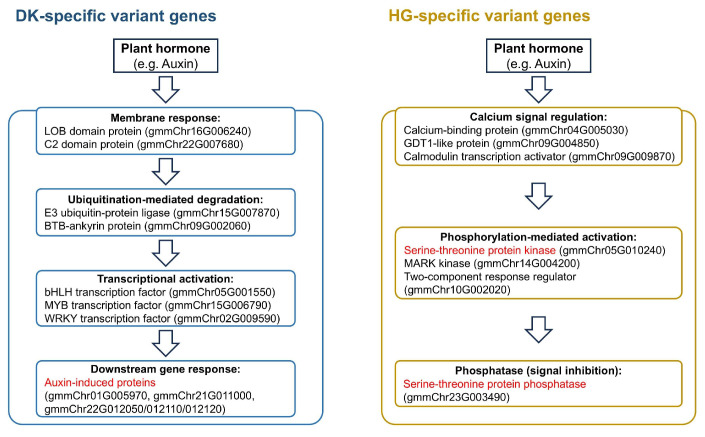
The signaling pathways regulated by variant-related genes. The frames highlighted the key sample-specific variants in *Phyllanthus emblica* cv. ‘Dongkeng’ (DK) and ‘Hongguang’ (HG) involved in plant hormone signal transduction, and the red color highlighted the key variant genes.

In the “Plant hormone signal transduction” pathway, 36 and 33 variant-containing genes were identified in the DK and HG samples, respectively (Table S4, Figure 3). Further KEGG functional profiling revealed that these sample-specific variants were mapped to distinct regulatory nodes within the hormonal signal transduction pathway (Figure 4). Specifically, the variants in the DK individual were annotated across the canonical auxin signaling axis, spanning membrane responses (LOB and C2 domain proteins), ubiquitin-mediated degradation (E3 ubiquitin-protein ligase and BTB-ankyrin protein), transcriptional activation (bHLH, MYB, and WRKY transcription factors), and downstream auxin-induced proteins (Figure 4). Notably, five specific auxin-induced protein genes were found to harbor mutations, which were downstream components previously reported to modulate hormonal signaling efficiency and enhance environmental tolerance [26]. Conversely, the variant genes in the HG sample were predominantly annotated with tuning signaling velocity and homeostatic feedback, notably targeting calcium signal regulation (calcium-binding protein, GDT1-like protein, calmodulin transcription activator), phosphorylation-mediated activation (serine-threonine protein kinase, MARK kinase, two-component response regulator), and signal inhibition (serine-threonine protein phosphatase) (Figure 4). As previously reported, protein phosphatases antagonized protein kinases to coordinate hormonal and stress responses, altering both concurrently fine-tuned signaling pulses [27]. Given the limitation of the two-sample comparison, extensive physiological validation will be essential to verify whether these mutational differences truly translate into optimized environmental fitness for these lineages.

### 3.3. Comparative Chloroplast Genomics and Mutational Landscapes of Two Phyllanthus emblica Genomes

**Figure 5:**
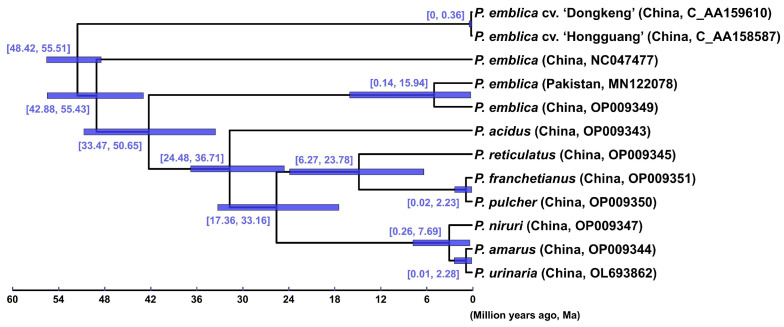
The phylogenetic time tree and divergence time estimation of *Phyllanthus emblica* cv. ‘Dongkeng’ (DK) and ‘Hongguang’ (HG) with seven related *Phyllanthus* species based on whole chloroplast genomes. The blue bars indicated the 95% highest posterior density (HPD) intervals of divergence times.

Through de novo assembly, the complete chloroplast genomes of the two *P. emblica* individuals, DK and HG, were obtained, with lengths of 156,231 bp (13,735.58×) and 156,232 bp (10,701.86×), respectively (Table S5). Notably, the relative coverage calculated from high-throughput sequencing was highly consistent with the qPCR verification, indicating that the relative chloroplast DNA copy number in sample DK was significantly higher than that in HG (*p* < 0.05) (Table S5). It was previously reported that light-stimulated instability can lead to a rapid decline in chloroplast DNA during plant plastid development [28]. The phylogenetic time tree consisting of the 12 chloroplast genomes indicated that the divergence of *P. emblica* occurred relatively early within the *Phyllanthus* genus (33.47–55.51 Ma, Eocene to Oligocene epochs) (Figure 5). Crucially, the lineage leading to the *P. emblica* samples DK and HG analyzed in this study exhibited an early divergence history (48.42–55.51 Ma), yet their individual chloroplast genomes showed almost no phylogenetic divergence between them (Figure 5). Consistent with geographical distributions, the DK and HG samples exhibited a closer phylogenetic relationship with the Chinese *P. emblica* (NC047477) than with the Pakistani accession (MN122078) [8], reflecting highly localized maternal genetic backgrounds.

**Figure 6:**
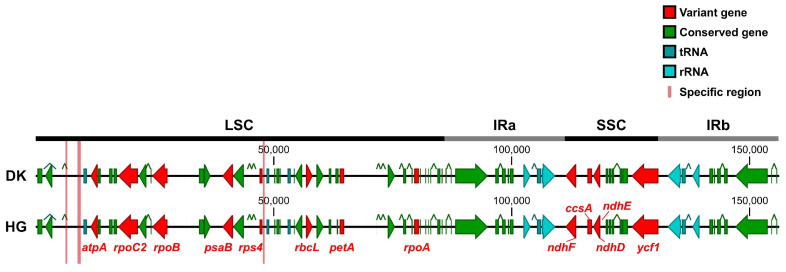
The distribution of chloroplast gene variants of *Phyllanthus emblica* cv. ‘Dongkeng’ (DK) and ‘Hongguang’ (HG). The four major regions (large single-copy (LSC), small single-copy (SSC), and two inverted repeats (IRa and IRb)), four categories of annotated genes (variant genes, conserved genes, tRNAs, rRNAs), specific regions in comparison to the chromosome were indicated.

Comparative analysis of chloroplast genome variants against the reference genome (NC047477) revealed identical variation patterns in both DK and HG individuals (Figure 6, Table S6), indicating that they shared a common maternal lineage. In addition to the 118 highly conserved genes, a total of 13 variant genes were identified, all of which were distributed exclusively within the large single-copy (LSC) and small single-copy (SSC) regions (Figure 6). Functional classification revealed that a significant proportion of the localized variants occurred within genes directly governing photosynthetic processes, specifically encompassing a core reaction center subunit (*psaB*), a cytochrome complex component (*petA*), and multiple subunits of the NAD(P)H dehydrogenase complex (*ndhD*, *ndhE*, and *ndhF*) (Table S6). Given that the *ndh* gene cluster mediated cyclic electron transport (CET) and was essential for photosynthetic stability under fluctuating environments [29], these synchronized variations provided a shared genomic baseline potentially associated with the micro-evolutionary adaptation in both the DK and HG samples. The remaining variant genes were primarily classified into molecular machinery responsible for chloroplastic genetic information expression and cellular maintenance, notably clustering within the core subunits of the RNA polymerase complex, including *rpoA*, *rpoB*, and *rpoC2* (Table S6). Previously, the plastid *rpoC1* locus was established as a reliable DNA barcode to discriminate various *Phyllanthus* species [30]. Although the analyzed DK and HG samples exhibited a close phylogenetic affinity with other *P. emblica* germplasms, the unique mutations within their *rpoA*, *rpoB*, and *rpoC2* clusters offered candidate markers for resolving fine-scale genetic differentiation among closely related lineages. Importantly, these genomic differences were derived from two individuals, and broader population-level sequencing is required to confirm them as fixed lineage-specific markers.

## 4. Conclusions

Overall, this study provided a comparative genomic framework for two sequenced individuals representing the *Phyllanthus emblica* cultivars, ‘Dongkeng’ (DK) and ‘Hongguang’ (HG), uncovering distinct nuclear and chloroplast variation patterns potentially associated with their evolutionary adaptation. Nuclear phylogenetic reconstruction based on ITS and *PHYC* sequences authenticated both samples within the *P. emblica* lineage, demonstrating that the single-copy *PHYC* gene offered superior resolution for subgeneric classification. Notably, the sequenced HG individual exhibited a relatively independent genetic position, highlighting its unique genetic identity. Genome-wide variant profiling against the wild-type reference revealed substantial polymorphisms; based on KEGG functional annotations, sample-specific mutations mapped to different nodes of the plant hormone signal transduction pathway. DK variants primarily spanned the canonical auxin signaling axis, whereas HG variants mapped to pathways regulating calcium signaling and phosphorylation-mediated homeostatic feedback, offering candidate genetic loci for future studies on their divergent growth habits. Furthermore, comparative chloroplast genomics demonstrated that despite sharing a common maternal lineage and identical mutational landscapes, the DK sample exhibited a higher relative chloroplast DNA copy number than HG. The identified localized variants within key photosynthetic genes (*psaB*, *petA*, and *ndh* cluster) and core RNA polymerase subunits (*rpoA*, *rpoB*, and *rpoC2*) offered candidate fine-scale molecular markers for cultivar differentiation. In conclusion, these findings provided a preliminary genomic exploration of the differences between *P. emblica* specific lineages and offered candidate genomic resources for future marker-assisted breeding. Importantly, these functional and evolutionary insights were derived from two individuals, necessitating broader population-level sequencing and physiological validation to confirm them as fixed lineage-specific markers.

## Data Availability

Sequence data are available at the National Genomics Data Center (NGDC) BioProject database with accession PRJCA057651; code files and photos are available at the Figshare with DOI 10.6084/m9.figshare.32625522.

## Supplementary Materials

The following supporting information can be downloaded at https://www.mdpi.com/article/10.3390/life16071138/s1: Table S1: Information for the internal transcribed spacer (ITS) sequences of *Phyllanthus* species used in this study; Table S2: Information for the phytochrome C (*PHYC*) sequences of *Phyllanthus* species used in this study; Table S3: Summary of sample-specific variants in *Phyllanthus emblica* cultivars “Dongkeng” (DK) and “Hongguang” (HG) against the reference genome *P. emblica* ‘Yingyu’; Table S4: KEGG functional enrichment analysis of sample-specific variant genes in *Phyllanthus emblica* cultivars ‘Dongkeng’ (DK) and ‘Hongguang’ (HG); Table S5: Information for the chloroplast genome sequences of *Phyllanthus* species used in this study; Table S6: Chloroplast gene variants of *Phyllanthus emblica* cultivars ‘Dongkeng’ (DK) and ‘Hongguang’ (HG) against the reference of NC047477.

## Abbreviations

The following abbreviations are used in this manuscript:

HG	*Phyllanthus emblica* cv. ‘Hongguang’
DK	*Phyllanthus emblica* cv. ‘Dongkeng’
qPCR	Real-time quantitative PCR
ITS	Internal transcribed spacer
PHYC	phytochrome C
ML	Maximum likelihood
SNP	Single-nucleotide polymorphism
CDS	Coding sequence
