# Supplementary material for: Comparative Genomic Analysis of Two Phyllanthus emblica Genomes with Endemic and Widespread Cultivar Backgrounds
Source: Life (Basel). 2026 Jul 9;16(7):1138. doi: 10.3390/life16071138 (PMC13412785; doi:10.3390/life16071138)
Supplement: Supplementary file 1 [file life-16-01138-s001.zip › life-4418580-supplementary.pdf]

Table S1. Information for the internal transcribed spacer (ITS) sequences of *Phyllanthus* species used in this study.

No.	Accession	Species taxonomy	Cultivar	Geographic location	Length (bp)	Coverage (×)
1	MN915853	*P. annamensis*	-	Viet Nam	427	-
2	MN915854	*P. annamensis*	-	Viet Nam	427	-
3	AB550082	*P. emblica*	-	Thailand	426	-
4	AY830087	*P. emblica*	-	China	426	-
5	GU441832	*P. emblica*	-	China	426	-
6	HM236855	*P. emblica*	-	India	426	-
7	KF926380	*P. emblica*	Anand-2	India	426	-
8	KF926382	*P. emblica*	Chakaiya	India	426	-
9	KF926384	*P. emblica*	Kanchan	India	426	-
10	KF926385	*P. emblica*	Krishna	India	426	-
11	KF926386	*P. emblica*	NA-7	India	426	-
12	KF938920	*P. emblica*	-	India	426	-
13	KF938922	*P. emblica*	-	India	426	-
14	OM677795	*P. emblica*	-	India	426	-
15	PQ460257	*P. emblica*	-	India	426	-
16	MN915936	*P. geoffrayi*	-	Thailand	427	-
17	AB550100	*P. oxyphyllus*	-	Thailand	426	-
18	AY936719	*P. oxyphyllus*	-	Austria	426	-
19	MN916029	*P. phuquocensis*	-	Cambodia	427	-
20	MN916035	*P. racemosus*	-	Sri Lanka	426	-
21	C AA158584	*P. emblica*	Dongkeng	China	426	8,397.00
22	C AA158585	*P. emblica*	Hongguang	China	427	10,460.00

Table S2. Information for the phytochrome C (*PHYC*) sequences of *Phyllanthus* species used in this study.

No.	Accession	Species taxonomy	Cultivar	Geographic location	Length (bp)	Coverage (×)
1	MN904223	*P. annamensis*	-	Viet Nam	567	-
2	MN904224	*P. annamensis*	-	Viet Nam	567	-
3	FJ235343	*P. emblica*	-	Japan	567	-
4	GQ503434	*P. emblica*	-	Netherlands	567	-
5	MN904279	*P. emblica*	-	Myanmar	567	-
6	MN904315	*P. geoffrayi*	-	Thailand	567	-
7	MN904389	*P. oxyphyllus*	-	Singapore	567	-
8	MN904400	*P. phuquocensis*	-	Cambodia	567	-
9	MN904405	*P. racemosus*	-	Sri Lanka	567	-
10	C AA158586	*P. emblica*	Dongkeng	China	567	69.85
11	C AA158587	*P. emblica*	Hongguang	China	567	79.69

Table S3. Summary of sample-specific variants in *Phyllanthus emblica* cultivars 'Dongkeng' (DK) and 'Hongguang' (HG) against the reference genome *P. emblica* 'Yingyu'.

Item	No. in DK	No. in HG
Total variants	3,458,532	3,434,313
Non-coding variants	3,354,371 (96.99%)	3,331,254 (97.00%)
Coding variants	104,161 (3.01%)	103,059 (3.00%)

Table S4. KEGG functional enrichment analysis of sample-specific variant genes in *Phyllanthus emblica* cultivars 'Dongkeng' (DK) and 'Hongguang' (HG).

Pathway	No. of Genes (DK)	No. of Genes (HG)	Gene IDs
Plant hormone signal transduction	36	33	DK:gmmChr01G005970,gmmChr01G017100,gmmChr02G001550,gmmChr02G008080,gmmChr02G009590,gmmChr02G010330,gmmChr02G011410,gmmChr05G001550,gmmChr07G007140,gmmChr08G006570,gmmChr08G009770,gmmChr09G002060,gmmChr09G002070,gmmChr09G007580,gmmChr10G002030,gmmChr10G003660,gmmChr10G005540,gmmChr14G004950,gmmChr15G003060,gmmChr15G006790,gmmChr15G007870,gmmChr16G002340,gmmChr16G002350,gmmChr16G004690,gmmChr16G005830,gmmChr16G006240,gmmChr16G007120,gmmChr16G009950,gmmChr16G010760,gmmChr19G006100,gmmChr21G011000,gmmChr22G007680,gmmChr22G012050,gmmChr22G012110,gmmChr22G012120,gmmChr24G006690<br>HG:gmmChr09G009870,gmmChr14G008030,gmmChr14G008040,gmmChr24G008980,gmmChr01G006180,gmmChr01G012330,gmmChr04G003610,gmmChr04G003860,gmmChr04G005030,gmmChr05G007900,gmmChr05G010240,gmmChr07G010220,gmmChr09G004620,gmmChr09G004850,gmmChr09G009860,gmmChr10G002020,gmmChr10G008870,gmmChr10G010440,gmmChr10G011140,gmmChr14G004200,gmmChr14G008020,gmmChr14G008410,gmmChr16G002110,gmmChr16G003170,gmmChr18G002970,gmmChr18G008690,gmmChr19G002690,gmmChr19G007050,gmmChr21G010960,gmmChr22G007280,gmmChr22G012060,gmmChr22G0
Nucleocytoplasmic transport	16	18	DK:gmmChr04G005240,gmmChr19G001250,gmmChr01G005670,gmmChr01G010720,gmmChr02G002450,gmmChr05G009490,gmmChr05G009810,gmmChr07G006660,gmmChr07G006690,gmmChr10G004380,gmmChr15G002880,gmmChr15G009950,gmmChr19G001240,gmmChr19G010990,gmmChr24G008360,gmmChr24G008410<br>HG:gmmChr10G010900,gmmChr01G012450,gmmChr02G000790,gmmChr02G004820,gmmChr02G006520,gmmChr05G006740,gmmChr06G009630,gmmChr07G006700,gmmChr08G004070,gmmChr08G008510,gmmChr10G010890,gmmChr14G007830,gmmChr15G006570,gmmChr16G002900,gmmChr18G001900,gmmChr18G006850,gmmChr18G007870,gmmChr19G005060
MAPK signaling pathway	17	13	DK:gmmChr02G001550,gmmChr07G007140,gmmChr07G007750,gmmChr07G008580,gmmChr08G006570,gmmChr08G006670,gmmChr08G009310,gmmChr10G001380,gmmChr14G004210,gmmChr16G005830,gmmChr16G006240,gmmChr18G007320,gmmChr18G007770,gmmChr19G003150,gmmChr19G006100,gmmChr19G010450,gmmChr24G006690<br>HG:gmmChr04G009770,gmmChr08G008390,gmmChr01G006180,gmmChr05G010240,gmmChr06G010240,gmmChr06G010750,gmmChr10G011450,gmmChr14G004200,gmmChr17G008130,gmmChr17G008560,gmmChr19G002690,gmmChr21G009910,gmmChr23G003490
Plant-pathogen interaction	15	15	DK:gmmChr10G004590,gmmChr01G006640,gmmChr01G016890,gmmChr02G001550,gmmChr04G002900,gmmChr08G009310,gmmChr10G001380,gmmChr10G002500,gmmChr10G004580,gmmChr15G002910,gmmChr16G002100,gmmChr16G004950,gmmChr16G006510,gmmChr18G008600,gmmChr23G006640<br>HG:gmmChr14G009750,gmmChr08G004790,gmmChr09G003540,gmmChr10G004550,gmmChr10G011400,gmmChr14G009740,gmmChr16G010050,gmmChr18G002730,gmmChr19G008280,gmmChr23G003090,gmmChr23G008360,gmmChr23G008450,gmmChr24G002090,gmmChr24G002960,gmmChr24G010670
Lysosome	12	17	DK:gmmChr01G011060,gmmChr04G008400,gmmChr05G009820,gmmChr09G003190,gmmChr09G008490,gmmChr10G004020,gmmChr14G003910,gmmChr16G009860,gmmChr17G010050,gmmChr18G009050,gmmChr21G010390,gmmChr23G006410<br>HG:gmmChr01G017180,gmmChr01G017190,gmmChr01G017170,gmmChr02G009270,gmmChr05G002960,gmmChr05G002980,gmmChr05G007510,gmmChr06G007720,gmmChr09G005020,gmmChr09G007870,gmmChr10G001560,gmmChr15G005750,gmmChr16G010710,gmmChr19G002960,gmmChr23G007200,gmmChr23G007840,gmmChr24G007730
Phagosome	12	12	DK:gmmChr01G012200,gmmChr04G008400,gmmChr09G003190,gmmChr10G004020,gmmChr16G009860,gmmChr17G001090,gmmChr17G004120,gmmChr17G007840,gmmChr18G001330,gmmChr23G006410,gmmChr23G010280,gmmChr24G007000<br>HG:gmmChr02G004490,gmmChr05G002960,gmmChr05G002980,gmmChr08G007500,gmmChr08G010050,gmmChr09G008220,gmmChr16G004290,gmmChr17G006070,gmmChr17G007480,gmmChr22G011290,gmmChr22G011530,gmmChr23G005330
Ubiquitin mediated proteolysis	9	9	DK:gmmChr17G002090,gmmChr07G010370,gmmChr10G001310,gmmChr14G005570,gmmChr17G002080,gmmChr17G006590,gmmChr18G000820,gmmChr22G011560,gmmChr24G008560<br>HG:gmmChr01G008270,gmmChr04G002000,gmmChr04G007210,gmmChr04G009060,gmmChr06G009230,gmmChr10G007870,gmmChr17G001140,gmmChr21G010520,gmmChr22G006030
RNA degradation	9	6	DK:gmmChr10G004210,gmmChr02G001160,gmmChr10G004200,gmmChr10G005350,gmmChr10G006720,gmmChr10G006730,gmmChr15G006670,gmmChr17G009260,gmmChr23G003810<br>HG:gmmChr02G008420,gmmChr10G005070,gmmChr14G004780,gmmChr16G008810,gmmChr19G008260,gmmChr19G008290
Peroxisome	6	7	DK:gmmChr01G015680,gmmChr02G008330,gmmChr18G002520,gmmChr19G007240,gmmChr24G007270,gmmChr24G011040<br>HG:gmmChr04G009770,gmmChr02G006490,gmmChr06G008890,gmmChr07G005490,gmmChr14G005520,gmmChr19G004320,gmmChr24G003070
Cellular senescence	6	4	DK:gmmChr10G001380,gmmChr14G003940,gmmChr14G008610,gmmChr15G003780,gmmChr19G001660,gmmChr23G009850<br>HG:gmmChr04G008700,gmmChr08G004980,gmmChr10G005070,gmmChr24G003760

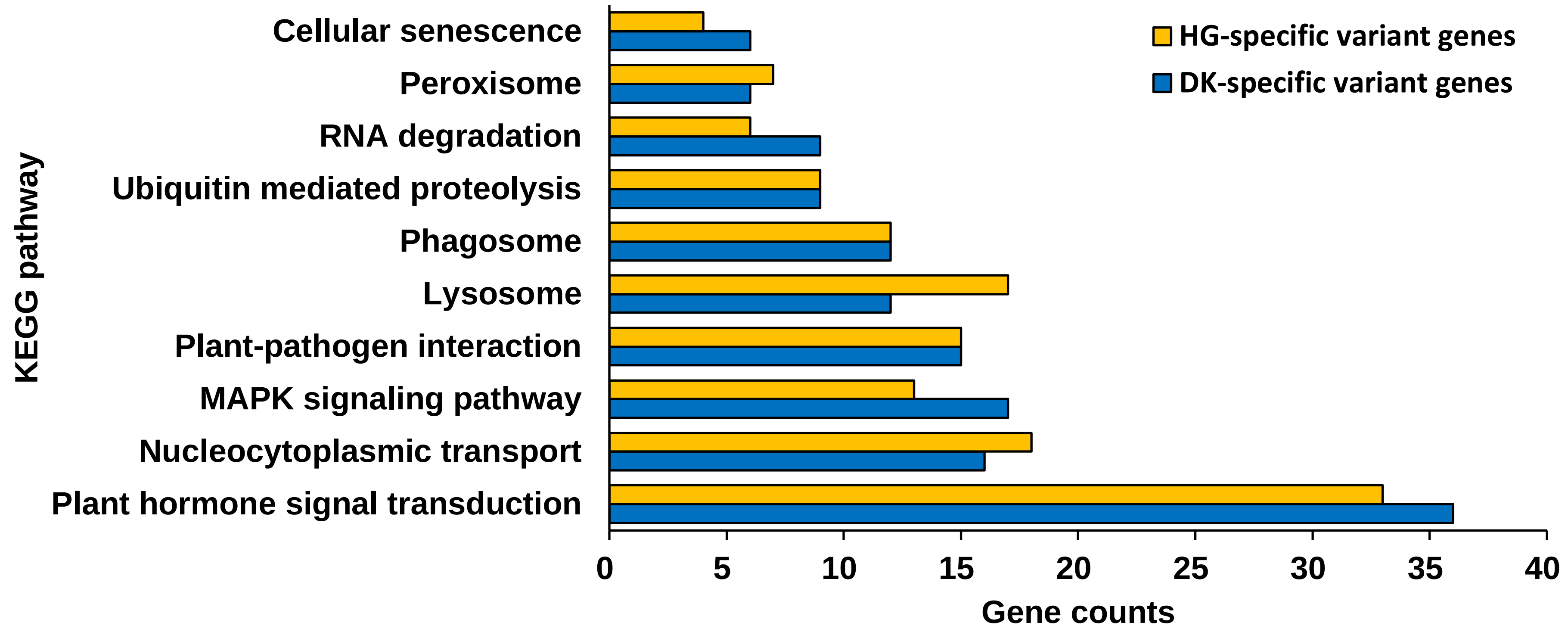

Table S5. Information for the chloroplast genome sequences of *Phyllanthus* species used in this study.

No.	Accession	Species taxonomy	Cultivar	Geographic location	Length (bp)	Coverage (×)	Relative copies (Relative coverage)	Relative copies ($2^{-\Delta Ct}$)
1	OP009343	*P. acidus*	-	China	156,331	-	-	-
2	OP009344	*P. amarus*	-	China	155,790	-	-	-
3	NC047477	*P. emblica*	-	China	156,208	-	-	-
4	MN122078	*P. emblica*	-	Pakistan	156,447	-	-	-
5	OP009349	*P. emblica*	-	China	155,841	-	-	-
6	OP009351	*P. franchetianus*	-	China	155,598	-	-	-
7	OP009347	*P. niruri*	-	China	155,900	-	-	-
8	OP009350	*P. pulcher*	-	China	155,589	-	-	-
9	OP009345	*P. reticulatus*	-	China	156,610	-	-	-
10	OL693862	*P. urinaria*	-	China	157,673	-	-	-
11	C AA159610	*P. emblica*	Dongkeng	China	156,231	13,735.58	196.64	219.01 ± 6.78
12	C AA159611	*P. emblica*	Hongguang	China	156,232	10,701.86	134.29	165.36 ± 1.88

Table S6. Chloroplast gene variants of *Phyllanthus emblica* cultivars 'Dongkeng' (DK) and 'Hongguang' (HG) against the reference of NC047477.

**No.**	**Variant position / Gene length (bp)**	**Reference**	**Variant**	**Locus tag**	**Gene**	**Product**
1	42/1506	G	A	HJE39_pgp080	*atpA*	ATP synthase subunit alpha
2	3601/4158	T	C	HJE39_pgp075	*rpoC2*	DNA-directed RNA polymerase subunit beta"
3	1864/4158	C	G	HJE39_pgp075	*rpoC2*	DNA-directed RNA polymerase subunit beta"
4	1504/3213	A	G	HJE39_pgp073	*rpoB*	DNA-directed RNA polymerase subunit beta
5	1725/2205	A	T	HJE39_pgp066	*psaB*	Photosystem I P700 chlorophyll a apoprotein A2
6	242/606	A	T	HJE39_pgp063	*rps4*	Small ribosomal subunit protein uS4c
7	1299/1449	G	A	HJE39_pgp057	*rbcL*	Ribulose bisphosphate carboxylase large chain
8	219/963	T	G	HJE39_pgp052	*petA*	Cytochrome f
9	909/1029	G	T	HJE39_pgp032	*rpoA*	DNA-directed RNA polymerase subunit alpha
10	2237/2244	TAAA	T	HJE39_pgp018	*ndhF*	NAD(P)H-quinone oxidoreductase subunit 5
11	182/966	C	T	HJE39_pgp016	*ccsA*	Cytochrome c biogenesis protein
12	1395/1521	T	C	HJE39_pgp015	*ndhD*	NAD(P)H-quinone oxidoreductase chain 4
13	261/306	A	G	HJE39_pgp013	*ndhE*	NAD(P)H-quinone oxidoreductase subunit 4L
14	2846/5640	C	T	HJE39_pgp007	*ycf1*	Hypothetical chloroplast protein
15	2730/5640	C	A	HJE39_pgp007	*ycf1*	Hypothetical chloroplast protein
16	369/5640	A	G	HJE39_pgp007	*ycf1*	Hypothetical chloroplast protein
